# Characterization of the Cafeteria Diet as Simulation of the Human Western Diet and Its Impact on the Lipidomic Profile and Gut Microbiota in Obese Rats

**DOI:** 10.3390/nu15010086

**Published:** 2022-12-24

**Authors:** Ana Laura de la Garza, Alejandra Mayela Martínez-Tamez, Anael Mellado-Negrete, Sofía Arjonilla-Becerra, Gloria Itzel Peña-Vázquez, Luis Martín Marín-Obispo, Carmen Hernández-Brenes

**Affiliations:** 1 Centro de Investigación en Nutrición y Salud Pública, Facultad de Salud Pública y Nutrición, Universidad Autonoma de Nuevo Leon, Monterrey 64460, Mexico; 2 Unidad de Nutrición, Centro de Investigación y Desarrollo en Ciencias de la Salud, Universidad Autonoma de Nuevo Leon, Monterrey 64460, Mexico; 3 Tecnologico de Monterrey, Escuela de Ingeniería y Ciencias, Campus Monterrey, Monterrey 64849, Mexico; 4 Integrative Biology Unit, The Institute for Obesity Research, Tecnologico de Monterrey, Monterrey 64849, Mexico

**Keywords:** obesity, *Bacteroidetes*, *Firmicutes*, *TM7*, capric acid, oleic acid, α-linolenic acid

## Abstract

The obesity pandemic has been strongly associated with the Western diet, characterized by the consumption of ultra-processed foods. The Western lifestyle causes gut dysbiosis leading to impaired fatty acid metabolism. Therefore, this study aimed to evaluate shifts in gut microbiota and correlate these with serum fatty acid profiles in male Wistar rats fed a cafeteria diet. Ten male rats were fed with standard diet (CTL, *n* = 5) and cafeteria diet (CAF, *n* = 5) for fifteen weeks. Body weight and food intake were recorded once and three times per week, respectively. At the end of the study, fresh fecal samples were collected, tissues were removed, and serum samples were obtained for further analyses. Gut microbiota was analyzed by sequencing the V3-V4 region of 16S rRNA gene. Serum fatty acid profiles were fractioned and quantified via gas chromatography. The CAF diet induced an obese phenotype accompanied by impaired serum fatty acids, finding significantly higher proportions of total saturated fatty acids (SFAs) and C20:3 *n*-6, and lower C18:1 *n*-7 and C18:3 *n*-3 in the phospholipid (PL) fraction. Furthermore, circulating C10:0, total *n*-3 and *n*-7 decreased and total monounsaturated fatty acids (MUFAs), including oleic acid C18:1 *n*-9, increased in the cholesterol ester (CE) fraction. The obesity metabotype may be mediated by gut dysbiosis caused by a cafeteria diet rich in C16:0, C18:0, C18:1 *n*-9 and C18:2 *n*-6 fatty acids resulting in a 34:1 omega-6/omega-3 ratio. Therefore, circulating C10:0 was associated with several genera bacteria such as *Prevotella* (positive) and *Anaerotruncus* (negative). Two classes of *Firmicutes*, *Bacilli* and *Erysipelotrichi*, were positively correlated with PL- C20:3 *n*-6 and CE- 18:1 *n*-9, respectively. *TM7* and *Bacteroidetes* were inversely correlated with PL-SFAs and CE- 18:2 *n*-6, respectively.

## 1. Introduction

Nowadays, globalization has fostered a transformation towards a more industrialized food environment [1]. Lifestyle and environment can influence ultra-processed food choices that consist of industrial formulations rich in sodium, added sugar and fats, among other substances such as preservatives, artificial colors and artificial flavors [2]. This has led to an obesity epidemic resulting from a prolonged imbalance between energy intake and expenditure caused by a complex multifactorial interaction [3]. To explore the mechanisms involved in the development of obesity, animal models have been used to simulate the common cause of human obesity, employing hypercaloric diets such as high-fat and high-sugar diets [4]. However, these diets do not adequately replicate the Western dietary pattern [5]. Meanwhile, cafeteria (CAF) diet handmade with ultra-processed foods has been used successfully in diet-induced obesity (DIO) studies [5]. Regarding the number of food products used in the CAF diet, there is a variety from three to ninety-six according to a review published by Lalanza et al. [5], the mean being between eight and nine food products. Secondly, some researchers interchange the use of ultra-processed foods throughout the experiment; however, others use a standardized artisanal diet that contains mainly cookies, fried potatoes, chocolate and animal fat such as bacon and pork pate, among other regional products [5,6].

It is recommended that sweet and salty foods be used, that is, energy-dense and highly palatable foods that lead to hyperphagia mimicking the human Western diet [7]. One characteristic of the Western diet is that it contains an excessive amount of omega-6 polyunsaturated fatty acids (*n*-6 PUFAs). PUFAs are characterized by more than one carbon–carbon double bond and have either cis or trans configuration in their carbon chain [8]. Omega-6 and omega-3 PUFAs are important components of cell membranes and precursors to other substances in the body involved in the inflammatory response. Furthermore, they are essential fatty acids that must be consumed by humans due to the lack of endogenous enzyme for desaturation [9]. Linoleic acid (LA) C18:2 *n*-6 and α-linolenic acid (ALA) C18:3 *n*-3 are fatty acids that the human body cannot produce. Moreover, due to a metabolic competition between both fatty acids, an increase in the consumption of *n*-6 LA can reduce the metabolism of *n*-3 ALA, which increases the risk of cardiovascular diseases [9]. The Western diet contains high *n*-6 PUFA and low *n*-3 PUFA levels, resulting in a 20:1 *n*-6/*n*-3 ratio [10]. High *n*-6 and low *n*-3 diet has been reported to induce proinflammatory conditions and increase intestinal permeability leading to microbial dysbiosis [11]. Dysbiosis is the imbalance of microorganisms that occurs in the intestine, giving rise to alterations in the host physiology. Dysbiosis is characterized by a decrease in bacterial diversity, as well as changes in the relative abundance of the main phyla. Particularly, studies in animal and human obesity models have shown intestinal dysbiosis through changes in the *Firmicutes*/*Bacteroidetes* ratio [12,13].

The obesity phenotype, both in rodents and humans, is characterized by the predominance of the gut bacterial phylum *Firmicutes*, increasing the *Firmicutes*/*Bacteroidetes* ratio [13]. However, this proportion has become controversial because various studies have found an increase in the relative abundance of *Bacteroidetes* over *Firmicutes*, causing a dysbiosis associated with some diseases [13]. Thereby, recently research has deepened into the study of different taxonomic levels, such as bacterial families and genera with specific metabolic activities. For example, the decrease in the relative abundance of *Prevotella*, a genus of the *Bacteroidetes* phylum, has been associated with Western diet consumption, causing intestinal permeability and low-grade inflammation [14]. In addition, it has been seen that shifts in gut microbiota can lead to an obesity-associated metabotype due to alterations in the host–microbiome lipid co-metabolism [15]. Therefore, this study aimed to analyze the fatty acid content of the cafeteria diet and evaluate the effect on gut microbiota and serum fatty acid profiles in diet-induced obesity rats.

## 2. Materials and Methods

### 2.1. Diets

Rodent Lab Chow Diet 5001 was purchased from “Círculo ADN” S.A. de C.V. company (Mexico City, Mexico). The diet contains 30% of energy from protein, 57% from carbohydrates and 13% from fat by dry weight (3.35 kcal/g). Regarding the nutritional composition of fats, the diet includes 1.25% linoleic acid, 0.12% linolenic acid, 0.02% arachidonic acid, 0.31% omega-3 fatty acids, 1.39% total saturated fatty acids (SFAs) and 1.52% total monounsaturated fatty acids (MUFAs). The cafeteria diet (CAF) was handcrafted with a mix of fried potatoes, biscuits, bacon, standard chow diet, pork pate base and liquid chocolate on a 1:1:1:1:2:1 ratio. The diet contains: 12% of energy from protein, 39% from carbohydrates and 49% from fat by dry weight (3.72 kcal/g).

### 2.2. Fatty acid Composition of Cafeteria Diet

Lipid extracts of dry food samples (3 g) were obtained via the n-hexane extraction method [16]. Then, the lipid extracts were dried under vacuum in a rotary evaporator system (Büchi Rotavapor R-215, Büchi, Flawil, Switzerland) and the total lipid content was determined gravimetrically. The recovered lipids were transferred to vials and stored at −80 °C for fatty acid (FA) analysis.

To produce fatty methyl esters (FAMEs), the food lipids (10 mg) were dissolved in a n-hexane-toluene mixture (750 mL, 2:1 *v*/*v*) and spiked with an internal quantification standard (glyceryl triundecanoate, TG-C11:0, 110 µg/mL) acquired from Nu Chek Prep Inc. (Elysian, MN, USA). The FAMEs were obtained using methyl alcohol-H2SO4 (1 mL, 93:7 *v*/*v*), as previously described by Castillo et al. [17].

The composition of the FAMEs was analyzed on an Agilent 8860 gas chromatography system (GC) equipped with a flame ionization detector (Agilent Technologies. Inc., Santa Clara, CA, USA) and a 100 m × 0.25 mm i.d., 0.2 mm film thickness fused-silica capillary column SP-2380 (Supelco Inc., Bellefonte, PA, USA). The injection samples (2 µL) were performed in split mode 1:15 ratio at 260 °C. The GC parameters and settings were set up according to the method of Castillo et al. [17]. The identification and quantification of FAs was carried out by comparing the retention times and peak areas of a standard mixture of FAMEs (GLC 566, Nu Chek Prep Inc., Elysian, MN, USA) to an internal standard, as explained by Castillo et al. [17]. The FA content was expressed as the percentage of dry matter and lipid content.

### 2.3. Animal Procedures

Ten 6-week-old male Wistar rats supplied from “Círculo ADN” S.A. de C.V. company (Mexico City, Mexico) underwent a microbiological study upon arrival at the Center for Research and Development in Health Sciences, as established by the Center’s biosafety committee. The rats were kept without performing procedures in a two-week acclimatization period and were housed individually in a temperature-controlled room (21–23 °C) under a 12:12 h light:dark cycle with ad libitum water and food access (standard chow diet). Animals were randomly distributed into two groups (*n* = 5 per group) and were assigned a standard chow diet (CTL group) or cafeteria diet (CAF group) for fifteen weeks.

Food intake was recorded three times per week and body weight once a week. The daily calorie intake and food efficiency were calculated as previously described by Etxeberria et al. [18].

Fresh fecal samples were gathered in 15 mL Falcon tubes one day before the endpoint of the experiment, early in the morning and after the overnight fasting period, via abdominal massage. Fecal samples were frozen at −80 °C for further analyses.

At the end of the study, rats were euthanized after an overnight fasting, and blood was collected from the trunk. Serum samples were obtained via centrifugation (2000× *g* for 15 min) and were stored at −80 °C until further analyses. White adipose tissue depots including mesenteric (mWAT), epididymal (eWAT), retroperitoneal (rWAT) and subcutaneous (sWAT), as well as brown adipose tissue (BAT), soleus and gastrocnemius muscles, the liver, and the spleen were collected, weighed and immediately frozen in liquid nitrogen. 

The research was conducted in agreement with the national and institutional guidelines of the Animal Research Bioethics Committee and was approved by the Ethics Committee of the Faculty of Public Health and Nutrition (CE 1/2022-01).

### 2.4. Fatty Acid Profiling of Serum Cholesterol and Phospholipids Fractions

The lipids of serum samples (150 µL) were extracted via a modification of the Folch method [17]. Serum lipids were fractioned in five sorts of lipids with dissimilar polarities; namely, cholesterol ester (CE), triglyceride (TG), mono and di-glyceride (MG+DG), free fatty acid (FFA) and phospholipid (PL) fractions, employing solid-phase extraction aminopropyl cartridges (500 mg, 3 mL, Bond Elut NH2, Agilent Technologies Inc., Santa Clara, CA, USA), as described by Castillo et al. (2020). Cholesteryl nonadecanoate (CE-C19:0, 450 µg/mL), from Nu Chek Prep Inc. (Elysian, MN, USA), and 1,2-Diheptadecanoyl-sn-glycerol-3-phos-phoryl-choline (PC-C17:0/C17:0, 300 µg/mL, Abcam, Cambridge, UK) were added as internal extraction standards. The CE and PL lipid fractions were selected for their characterization and were transmethylated to produce the FAMEs, as previously reported by Castillo et al. [17]. TG- C11:0 (110 µg/mL) was added to each fraction as an internal quantification standard. The FAMEs of CE and PL fractions were analyzed using an Agilent 8860 GC-FID. The identification and quantification of FAs were performed, as previously mentioned. The absolute concentration (µg/mL) of individual FAs in each fraction was normalized to the total amount of FAs and expressed as the relative percent concentration.

### 2.5. Gut Microbiota Analysis from Male Wistar Rats by Illumina Mi-Seq Sequencing

DNA from the fecal samples was extracted using the QIAamp DNA Stool Mini Kit (Qiagen, Hilden, Germany) according to the manufacturer’s instructions. DNA quality and quantity were evaluated using spectrophotometry (NanoDrop 8000, Thermo Scientific, Wilmington, DE, USA) and a Picogreen fluorometer, respectively. The V3 and V4 regions of the 16S rRNA gene were amplified using specific forward (5′TCGTCGGCAGCGTCAGATGTGTATAAGAGACAGCCTACGGGNGGCWGCAG 3′) and reverse primers (5′GTCTCGTGGGCTCGGAGATGTGTATAAGAGACAGGACTA CHVGGGTATCTAATCC 3′) containing the Illumina adapter overhang nucleotide sequences. PCRs were carried out using the following parameters: 3 min 95 °C pre-denaturation; followed by 25 amplification cycles: 95 °C for 30 s, 63 °C for 30 s, 72 °C for 30 s and 72 °C for 5 min. Amplicon size was analyzed via 3% agarose gel electrophoresis (PowerPac, Bio-Rad, Hercules, CA, USA) and amplicons were purified using AMPure XP. The indices of the Nextera XT kit were added via new PCR, following Illumina’s protocol [19].

DNA sequencing was performed using the Illumina MiSeq platform according to the protocol suggested by Illumina (16S metagenomic sequencing library preparation). At the end of the process, a file in FASTQ format was obtained for each sample that included the readings. The data obtained were subjected to quality control and analyzed using QIIME v.1.9. Sequences with 97% identity were grouped into operational taxonomic units (OTUs) and taxonomically assigned by comparison against the Greengenes database. The readings were assigned to the phylum, class, order, family and genus levels. In addition, the α-diversity (Shannon index) was determined.

### 2.6. Statistical Analysis

Statistical analyses were conducted using SPSS 25.0 (SPSS Inc., Chicago, IL, USA). Results are expressed as mean ± SEM. Normality of data distribution was obtained using the Shapiro–Wilk test and Levene’s test with a *p*-value cutoff of 0.05. For normally distributed data, a *t*-Student test for independent samples was performed to analyze the statistical differences between group means and its non-parametric equivalent, the Mann–Whitney U test was used for non-normally distributed data. The correlation between different variables was performed using the Pearson test or its non-parametric Spearman equivalent. A level of probability of *p* < 0.05 was set as statistically significant. Sample sizes can be found in the figure legends, where *n* represents the number of animals used in each analysis.

## 3. Results

### 3.1. Fatty Acid Profile of the Cafeteria Diet as a Model of a Western Diet

As shown in Table 1, fatty acid (FA) content in the cafeteria diet was evaluated using gas chromatography. Thus, of the total saturated fatty acids (SFA), palmitic acid C16:0 was found in greater proportion, while lignoceric acid C24:0, a very-long-chain fatty acid (VLCFA), was found in a lower proportion. Secondly, as expected, oleic acid C18:1 *n*-9 was the most abundant among monounsaturated fatty acids (MUFA), since it is found in animal fats and vegetable oils commonly used in the cafeteria diet. Regarding polyunsaturated fatty acids (PUFA), the total *n*-3 was 0.19 ± 0.00 g/100 g dry matter and total *n*-6 was 6.49 ± 0.14 g/100 g dry matter, resulting in a 34.04 ± 0.29 *n*-6/*n*-3 ratio. The most predominant PUFA was linoleic acid C18:2 *n*-6 (6.62 ± 0.14 g/100 g dry matter).

### 3.2. Effect of Cafeteria Diet on Body Weight and Biometric Parameters in Male Wistar Rats

**Figure 1:**
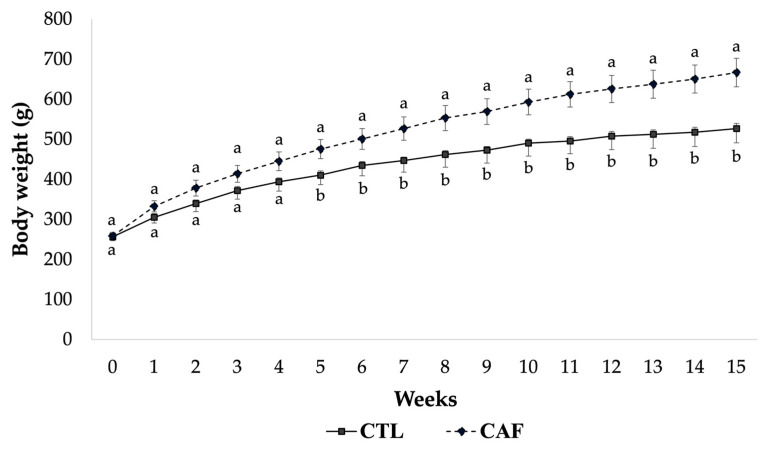
Effect of cafeteria diet on body weight during fifteen weeks in male Wistar rats. All the results are expressed as mean ± standard error (*n* = 5 per group). Statistical analysis was performed using Student’s *t* test for two independent samples. Mean values with different letters (a,b) show significant differences (*p* < 0.05). CTL, control group; CAF, cafeteria group.

In the present study, the CAF group had higher daily food intake (32.36 ± 1.23 g) as well as higher food efficiency (3.22 ± 0.12 g/100 kcal) compared with the CTL group (27.20 ± 0.42 g, 2.82 ± 0.03 g/100 kcal, respectively). Consistent with those findings, after five weeks eating the cafeteria diet, greater body weight gain was obtained in CAF group (475.76 ± 23.93 g) compared with the CTL group (410.48 ± 10.78 g) (*p* < 0.05). This difference was maintained throughout the study (Figure 1). However, although the CAF group indicated higher percentage of rWAT (3.29 ± 0.21 g/100 g BW) than the CTL group (2.87 ± 0.75 g/100 g BW), the difference was not statistically significant. Likewise, changes in the gastrocnemius muscle were observed between the CTL group (0.45 ± 0.03 g/100 g BW) and the CAF group (0.37 ± 0.02 g/100 g BW) without statistically significant differences (Table 2).

### 3.3. Shifts in Gut Microbiota after Eating Cafeteria Diet for 15 Weeks in Male Wistar Rats

**Figure 2:**
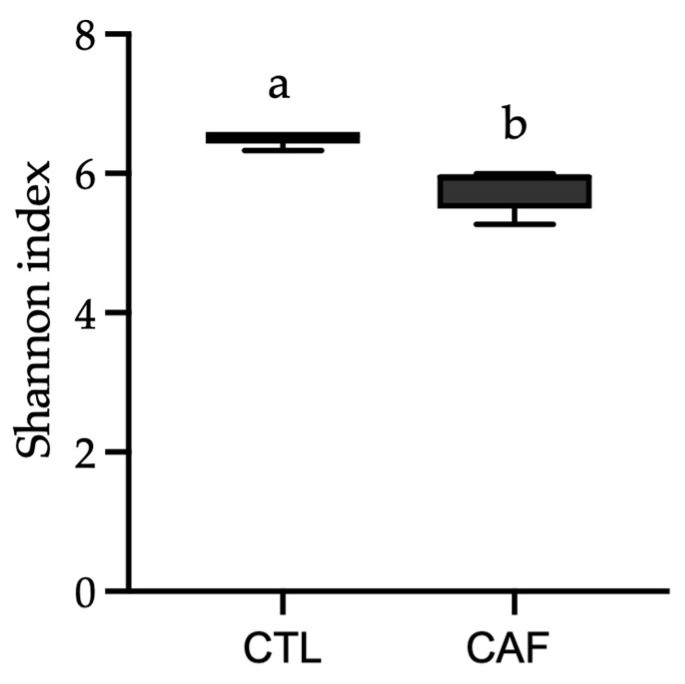
Gut dysbiosis in male Wistar rats fed cafeteria diet evaluated using the Shannon index. The results are expressed as mean ± standard deviation (*n* = 5). Statistical analysis was performed using Student’s *t* test for two independent samples. Mean values with different letters (a,b) show significant differences (*p* < 0.05). CTL: control group; CAF: cafeteria group.

**Figure 3:**
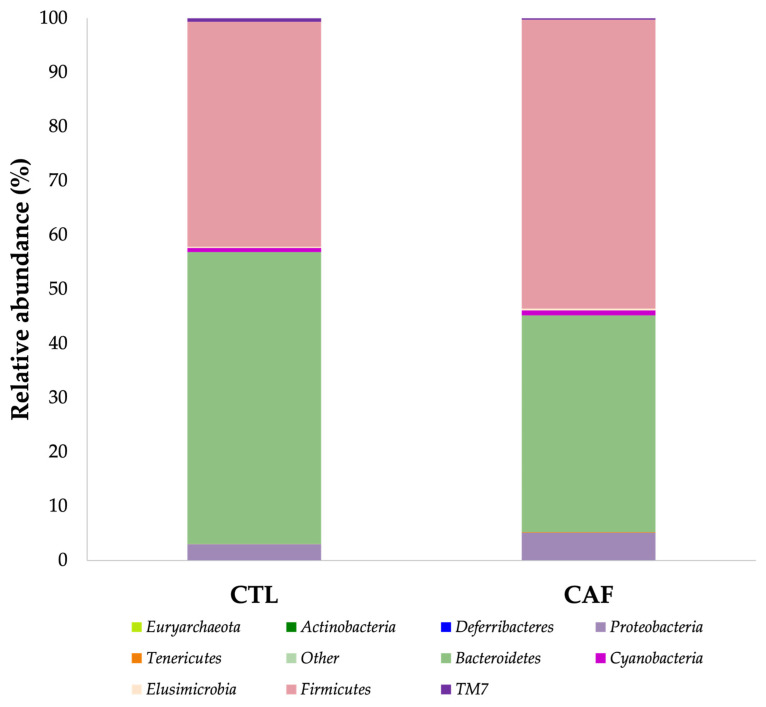
Relative abundance (>0.05%) of bacterial phyla in gut microbiota of male Wistar rats. CTL: control group; CAF: cafeteria group.

The gut microbiota α-diversity values were evaluated using Shannon’s diversity index. As shown in Figure 2, significant differences between the CTL group (6.51 ± 0.11) and the CAF group (5.77 ± 0.30) were found (*p* < 0.001). In fact, Figure 3 shows the relative abundance of 10 phyla found in both groups (>0.05%). The results indicate significant differences in the relative abundance of *Bacteroidetes* and *TM7* phyla between groups (*p* < 0.05 and *p* < 0.001, respectively). Moreover, the relationship between *Firmicutes* and *Bacteroidetes* relative abundance, expressed as *Firmicutes*/*Bacteroidetes* ratio (F/B ratio), was statistically significant between the CTL group (0.80 ± 0.11%) and the CAF group (1.41 ± 0.22%) (*p* < 0.05).

**Figure 4:**
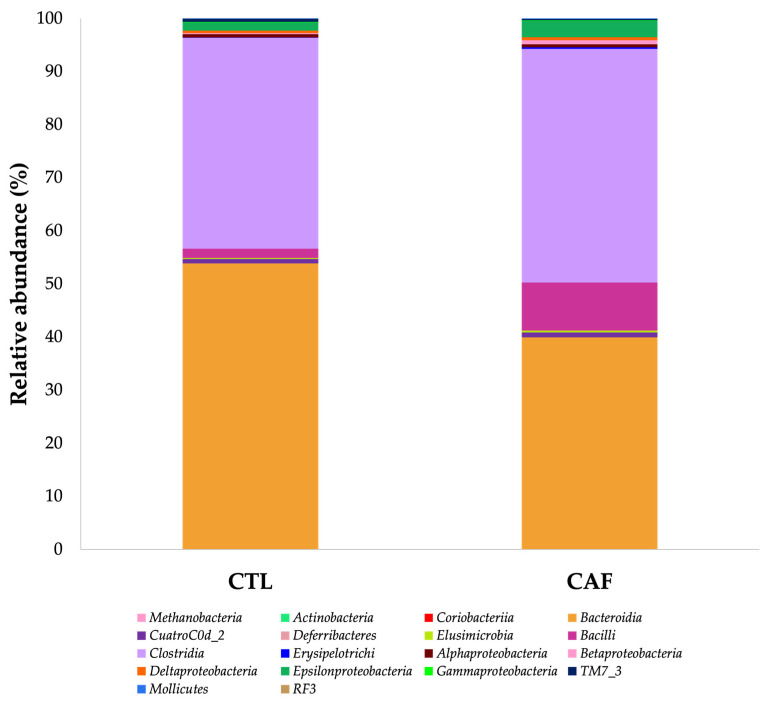
Relative abundance (>0.05%) of bacterial classes in gut microbiota of male Wistar rats. CTL: control group; CAF: cafeteria group.

The relative abundance (>0.05%) of bacterial classes in gut microbiota of male Wistar rats is represented in Figure 4. Thus, at the class taxonomic level, a significant decrease was found in the relative abundance of *Bacteroidia* (39.89 ± 3.54%) (Phylum: *Bacteroidetes*) and *TM7_3* (0.26 ± 0.02%) (Phylum: *TM7*) in the CAF group compared with 53.82 ± 3.68% and 0.64 ± 0.04% relative abundance in the CTL group, respectively (*p* < 0.05 and *p* < 0.001, respectively). Instead, a significant increase in the relative abundance of the *Bacilli* (9.06 ± 1.52%) and *Erysipelotrichi* (0.30 ± 0.08%) classes (Phylum: *Firmicutes*) was observed in the CAF group compared with the CTL group (1.75 ± 0.45% and 0.05 ± 0.01%) (*p* < 0.01 and *p* < 0.05, respectively).

At the order level, the CAF diet significantly decreased the relative abundance of *Bacteroidales* (39.89 ± 3.54%), *CW040* (0.26 ± 0.03%) and *Pasteurellales* (0.02 ± 0.01%) when compared with the CTL group (53.82 ± 3.68%, 0.64 ± 0.05% and 0.05 ± 0.01%, respectively) (Table 3), while the relative abundance of orders *Erysipelotrichales* (0.30 ± 0.08%) and *Lactobacillales* (8.82 ± 1.57%) and families *Erysipelotrichaceae* (0.34 ± 0.09%) and *Lactobacillaceae* (9.91 ± 1.70%) of the Firmicutes phylum increased in the CAF group compared with the CTL group (0.05 ± 0.01%, 1.48 ± 0.43%, 0.06 ± 0.01% and 1.65 ± 0.47%, respectively) (*p* < 0.05, *p* < 0.01, *p* < 0.05 and *p* < 0.01, respectively). In addition, at the family level, the relative abundance of *Prevotellaceae* (6.85 ± 1.84%) and *Rikenellaceae* (0.46 ± 0.08%) of the phylum *Bacteroidetes* decreased in the CAF group compared with the CTL group (16.50 ± 2.32% and 1.07 ± 0.21%) (*p* < 0.05).

The relative abundance of significantly altered genera among CTL and CAF groups were *Prevotella* (29.20 ± 3.68% versus 11.59 ± 3.26%) (Phylum: *Bacteroidetes*), *Aggregatibacter* (0.11 ± 0.02% versus 0.03 ± 0.01%) and *Sutterella* (0.46 ± 0.02% versus 1.45 ± 0.28%) (Phylum: *Proteobacteria*) (*p* < 0.01). The CAF diet induced an increase in some genera belonging to the phylum *Firmicutes*, such as *Allobaculum*, *Anaerotruncus*, *Eubacterium* and *Lactobacillus* (*p* < 0.05, *p* < 0.001, *p* < 0.05 and *p* < 0.01) (Table 3).

### 3.4. Lipid Metabolism Is Altered after Eating Cafeteria Diet for 15 Weeks in Male Wistar Rats

Ten serum phospholipid SFAs were identified and derived in four groups: medium-chain fatty acids (MCFA) C8:0, C10:0 and C12:0; long-chain fatty acids (LCFA) C14:0, C16:0 and C18:0; odd-chain fatty acids (OCFA) C15:0 and very-long-chain fatty acids (VLCFA) C20:0, C22:0 and C24:0 (Table 4). 

In general, neither PL-MUFAs nor PL-PUFAs nor the *n*-3, *n*-6, or *n*-9 series of unsaturated fatty acids (UFAs) demonstrated any significant differences between CTL and CAF groups. The fatty acids that contributed the highest proportion to the total PL-MUFAs were oleic acid C18:1 *n*-9 and vaccenic acid C18:1 *n*-7, demonstrating significant differences between groups only in the latter one (*p* < 0.05). Circulating PL α-linolenic acid (ALA) C18:3 *n*-3 and docosahexanoic acid C22:6 *n*-3 were lower in the CAF group (0.15 ± 0.03 µg/L % and 2.87 ± 0.18 µg/L %) compared with the CTL group (0.23 ± 0.03 µg/L % and 5.58 ± 1.19 µg/L %) (*p* < 0.05), while dihomo-γ-linoleic acid C20:3 *n*-6 was higher in the CAF group (1.91 ± 0.38 µg/L %) when compared with the CTL group (0.36 ± 0.06 µg/L %) (*p* < 0.05). The CTL group exhibited higher proportions of PL- total *n*-7 than the CAF group (*p* < 0.001) (Table 4).

In the CE fraction, C10:0 and C12:0 MCFAs were lower in the CAF group (4.34 ± 0.45 µg/L % and 5.55 ± 0.87 µg/L %) than the CTL group (9.08 ± 0.85 µg/L % and 8.67 ± 0.83 µg/L %) (*p* < 0.01 and *p* < 0.05, respectively). The CAF group exhibited higher proportions of total MUFAs than the CTL group, with PL- oleic acid C18:1 *n*-9 as the most abundant as expected with a concentration of 10.52 ± 1.83 µg/L % (*p* < 0.01). However, lower proportions of CE-PUFAs (C18:3 *n*-3, C20:3 *n*-6 and C20:4 *n*-6) were found in the CAF group compared with the CTL group (*p* < 0.01, *p* < 0.05 and *p* < 0.01, respectively). Likewise, CE- total *n*-3 and *n*-7 concentrations were lower in the CAF group compared with the CTL group (*p* < 0.05) (Table 5).

### 3.5. Alterations in the Host-Microbiome Lipid Co-Metabolism

**Figure 5:**
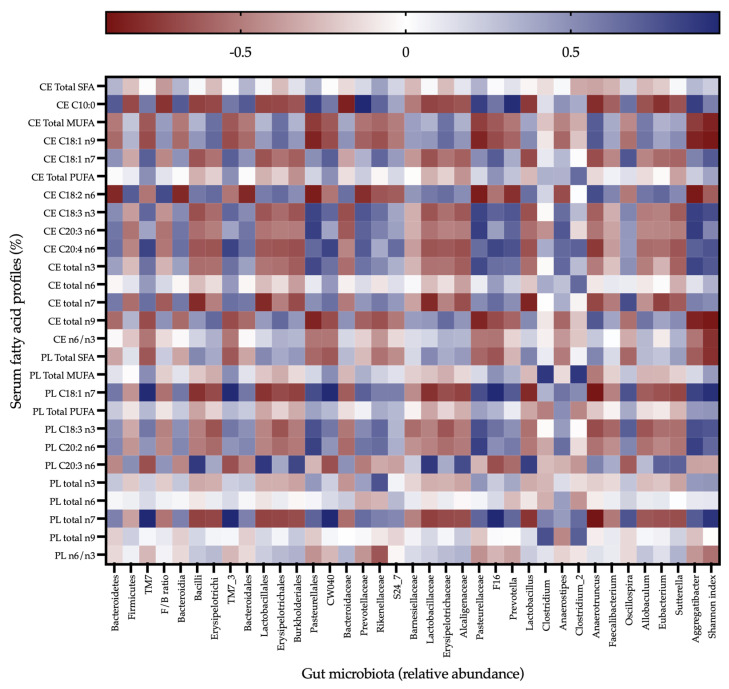
Correlation analysis between gut microbiome and serum metabolites assessed using Pearson’s correlation coefficient. Positive and negative correlation coefficients are colored blue and red, respectively. The intensity of the color represents the degree of correlation.

Figure 5 shows a heatmap of the Pearson’s correlation coefficients between PL and CE fatty acids with several gut microbiota at different taxonomic levels. The Shannon index, an indicator of bacterial diversity, was positively correlated with both PL- and CE- C18:1 *n*-7 and C18:3 *n*-3 fatty acids, as well as PL- C20:2 *n*-6, PL- total *n*-7, CE- C20:4 *n*-6, and CE- total *n*-3. However, PL- total SFAs, CE- total MUFAs, CE-C18:1 *n*-9, CE- total *n*-9 and CE- *n*-6/*n*-3 were inversely correlated with the Shannon index. 

Although no correlations were found between the relative abundance of Firmicutes and fatty acid levels, the class *Bacilli*, order *Lactobacillales*, family *Lactobacillaceae* and genus *Lactobacillus* were positively correlated with PL- C20:3 *n*-6. In addition, the genus *Eubacterium*, from the phylum *Firmicutes*, also demonstrated a positive correlation with homo-γ-linoleic acid. *Erysipelotrichi*, *Erysipelotrichales*, *Erysipelotrichaceae*, a class, order, and family of the phylum *Firmicutes*, respectively, were positively correlated with CE- C18:1 *n*-9. 

*Anaerotruncus*, a bacterial genus from the phylum *Firmicutes*, was positively correlated with CE- total MUFAs, CE- C18:1 *n*-9, CE- C18:2 *n*-6 and CE- total *n*-9, but inversely with CE- and PL- total *n*-7 and C18:1 *n*-7. Additionally, as expected, these MUFAs *n*-7 were negatively correlated with relative abundance of *Bacilli*, *Lactobacillales*, *Lactobacillaceae* and *Lactobacillus*.

*Pasteurellales*, *Pasteurellaceae* and *Aggregatibacter* (order, family and genus from the phylum *Proteobacteria*) were positively correlated with CE- and PL- C18:3 *n*-3, PL-C20:2 *n*-6, CE- C20:3 *n*-6 and CE- total *n*-3, and inversely correlated with CE- C18:2 *n*-6. Furthermore, these serum linoleic acid levels (CE- C18:2 *n*-6) were negatively correlated with phylum *Bacteroidetes*, class *Bacteroidia*, order *Bacteroidales*, family *Prevotellaceae* and genus *Prevotella*. *Prevotella* is a genus of Gram-negative bacteria positively correlated with PL- total *n*-7, PL- C18:1 *n*-7 and CE- C18:3 *n*-3.

Phylum *TM7*, class *TM7_3*, order *CW040* and family *F16* were positively correlated with PL- total *n*-7, PL- C18:1 *n*-7, CE- C18:1 *n*-7 and CE- C20:4 *n*-6, and inversely correlated with PL-SFAs.

Circulating CE C10:0 was associated with several genera of bacteria but had especially high correlations with *Prevotella* (positive) and *Anaerotruncus* (negative).

## 4. Discussion

In recent decades, the world has been involved in a more industrialized food environment, increasing the prevalence of obesity. Although obesity is a multifactorial disease, a factor associated with this pandemic is the high consumption of a Western diet [20]. The typical Western diet consists of a high-energy dietary pattern with large portions and a high content of fat, sugars, and sodium. Regarding fat content, it is noteworthy that the Western diet contains a high percentage of saturated fat and is characterized by a 20:1 *n*-6/*n*-3 ratio [10]. 

For the study of obesity, different animal models have been used, as well as different dietary patterns or hypercaloric diets such as high-fat, high-sugar and cafeteria diets [21,22]. However, a greater impact on the development of diet-induced obesity has been seen in models fed a cafeteria diet [5,21,23]. This may be because body weight is determined by a balance between energy intake and expenditure. In this sense, the cafeteria diet has been shown to be highly palatable, mimicking the Western diet, which contributes to weight gain and metabolic alterations associated with obesity [21]. In our study, similar to other authors [18,23], we found an increase in food efficiency in the CAF group compared to the CTL group. The foods used to make the cafeteria diet are ultra-processed and include hydrogenated fats and other ingredients such as artificial colors and flavors, making foods highly palatable, translating into a higher energy intake [2,24]. To understand the effect of the cafeteria diet on the development of obesity, we first characterized the fatty acid content of the food. As expected, the cafeteria diet is composed of a higher proportion of palmitic acid C16:0, stearic acid C18:0, oleic acid C18:1 *n*-9 and linoleic acid (LA) C18:2 *n*-6. Furthermore, it resulted in a 34:1 *n*-6/*n*-3 ratio. Palmitic acid is a long-chain saturated fatty acid (LCSFA) and the most abundant in animal products and other vegetable fats such as palm oil used in ultra-processed foods [25]. Palmitic acid has been associated with a pro-inflammatory response by activating Toll-like receptors- (TLRs) 2 and 4. Unlike lauric acid, a medium-chain saturated fatty acid (MCSFA), palmitic acid increased inflammation in male C57BL/6 mice fed a high-fat diet supplemented with 3% palmitic acid for 12 weeks [26]. However, although in our study circulating levels of palmitic acid were not found to be elevated in rats fed a cafeteria diet, other longer-chain or unsaturated fatty acids can be formed from it. In fact, palmitic acid can be converted to palmitoleic acid C16:1 *n*-7 by ∆-9 desaturation.

One study reported high levels of palmitoleic acid C16:1 *n*-7 and dihomo-γ-linolenic acid C20:3 *n*-6 in obese children, associating these fatty acids with an increased in metabolic risk factors [27]. Likewise, Bermúdez-Cardona et al. identified increased levels of PL- C16:1 *n*-7 and PL- C20:3 *n*-6 and decreased levels of PL- C18:2 *n*-6 in obese young adults with metabolic syndrome compared to adults with normal weight [28]. 

In our study, monounsaturated fatty acid C16:1 *n*-7 was found in both fractions of phospholipids (PL) and cholesterol esters (CE), without significant differences between groups. On the other hand, circulating PL- C20:3 *n*-6 was higher in the CAF group compared with the CTL group.

Furthermore, oleic acid is a monounsaturated fatty acid of the omega-9 series and widely present in vegetable oils such as safflower oil. The saturated form of this acid is stearic acid C18:0, which, in addition to being present in the Western diet, can also be synthesized from palmitic acid [29]. However, it has been seen that stearic acid is not absorbed in the intestine, so in our study no circulating levels in serum of stearic acid C18:0 were observed in Wistar rats fed a cafeteria diet. However, increased levels in serum of CE- C18:1 *n*-9 were found in the CAF group compared with the CTL group. On the other hand, although γ-linolenic acid (GLA) C18:3 *n*-6 increased in the CAF group, this was not significant. In a longitudinal study, biomarkers associated with metabolic disorders such as the HOMA index and insulin sensitivity were associated with the fatty acid profile. The authors reported that the higher circulating levels of C18:3 *n*-6 were correlated with lower insulin sensitivity. In contrast, circulating PL- C18:1 *n*-7 was positively associated with insulin sensitivity and pancreatic β-cell function [30].

Although in the cafeteria diet the content of capric acid C10:0 was not highlighted, the result of circulating C10:0 in rats fed cafeteria diet was striking, because although it is a MCSFA, it decreased in the CAF group compared with the CTL group. Capric acid is digested and absorbed in the stomach and is catalyzed by gastric lipase. In addition, this fatty acid increases energy expenditure, decreased adiposity and increases insulin sensitivity [12,31]. 

In fact, a study conducted by Li et al. demonstrated that C10:0 decreased weight gain and increased bile acid excretion in hypercholesterolemic C57BL/6J mice fed a diet high in medium-chain triglycerides [32].

Circulating fatty acids can come from the diet, as substrates for chemical reactions of metabolism, but they can also be derived from the product of bacteria that inhabit the host’s intestine, thus contributing to the pathogenesis of obesity. Thus, alterations in the lipidome may be related to diet-induced changes in bacterial composition and diversity [33].

Different studies have evaluated the effect of a diet rich in long-chain fatty acids (LCFA) in gut microbiota and the metabolic effects associated with gut dysbiosis [31]. For example, in a study with C57BL/6J mice fed a diet rich in palmitic acid, the authors observed increased body weight and increased relative abundance of Firmicutes and decreased *Bacteroidetes* [34]. The upregulation of *Firmicutes* abundance has been associated with impaired intestinal permeability, obesity and associated metabolic disturbances [35]. The phylum *Firmicutes* includes bacterial species with genes encoding enzymes involved in lipid and carbohydrate metabolism, so an increase in *Firmicutes*/*Bacteroidetes* (F/B) ratio has been associated with body weight gain and the development of obesity [13]. In our study, the F/B ratio was inversely correlated with CE C10:0. Gual-Grau et al. evaluated the effect of different hypercaloric diets on bacterial abundance and diversity, finding that the cafeteria diet decreased α-diversity more than the other obesogenic diets, compared to the standard diet, although the results were not statistically significant [21]. The decrease in bacterial diversity has been associated with obesity; in fact, in our study, we found a lower α-diversity in the CAF group compared with the CTL group. In the association between α-diversity and fatty acids, we found a positive correlation with seven fatty acids and a negative correlation with six of the total number of fatty acids that presented a significant difference between groups. The strongest association was with PL- C18:1 *n*-7, a MUFA that decreased in the CAF group. Furthermore, this fatty acid was positively associated with the relative abundance of *TM7*, *TM7_3*, *CW040* and *F16*. To date, little is known about the phylum *TM7*; however, a recent study found an association between *TM7* and adiposity markers [36]. In our study we found the opposite, as the relative abundance of *TM7* decreased significantly in the CAF group. Similar with our results, Hu et al. found a negative correlation between *TM7* and body weight [37]. Therefore, more studies on the phylum *TM7* are needed to understand its role in the obesity phenotype. What we can highlight in this study is that a relationship of *TM7* with total *n*-7 was found in the two fractions PL and CE and was involved in certain metabolic processes such as less fat storage in the liver, as well as improved insulin sensitivity [38]. In addition, the total *n*-7 was negatively correlated with the genus *Anaerotruncus*, from the phylum *Firmicutes*. Although there are few studies reporting the abundance of *Anaerotruncus*, Bortolin et al. observed an increase in this bacterial genus in male Wistar rats fed a cafeteria diet for 18 weeks. Interestingly, this genus has been associated with weight gain, metabolic diseases and intestinal permeability [39]. 

Other bacterial genera of the phylum *Firmicutes* that increased significantly in the CAF group were *Eubacterium*, *Lactobacillus* and *Allobaculum*. A study in 3-week-old BALB/c mice found an increase in *Eubacterium* abundance after two weeks of being fed a high-fat diet, but with 1:2 *n*-6/*n*-3 PUFAS, however, the authors did not report metabolic changes [40]. On the other hand, in a review on gut microbiota and obesity, the authors reported an increase in *Eubacterium* abundance in Japanese people with obesity [41]. Thus, the increased abundance of genus *Eubacterium* from the phylum *Firmicutes* could be associated with obesity, and in this study it was positively associated with PL- C20:3 *n*-6 and negatively with total *n*-7, as well as with C10:0. On the other hand, the genus *Prevotella*, from the phylum *Bacteroidetes*, has been associated with a diet rich in fiber [42]. Indeed, volunteers with risk of metabolic syndrome decreased *Prevotella* abundance after 4 weeks of consuming a high-fat *n*-6 PUFA diet, whereas the MUFA diet induced an increase in the relative abundance of *Prevotella*, lowering BMI [43]. In this sense, it has been seen that overweight adults with an upregulation of *Prevotella* abundance lose more weight than subjects with a downstream *Prevotella* abundance after consuming a high-fiber diet. In our study, a positive correlation of *Prevotella* abundance was found with C10:0 and PL- C18:1 *n*-7, and negative association with CE C18:2 *n*-6. 

In summary, the increased *Firmicutes*/*Bacteroidetes* ratio, including classes *Bacilli* and *Erysipelotrichi*, orders *Lactobacillales* and *Erysipelotrichales*, genera *Anaerotruncus*, *Eubacterium* and *Lactobacillus* (Phylum: *Firmicutes*), was positively associated with PL- C20:3 *n*-6 that was higher in the CAF group compared with the CTL group. On the other hand, relative abundance of the phylum *Bacteroidetes*, including class *Bacteroidia*, order *Bacteroidales*, family *Prevotellaceae*, and genus *Prevotella* significantly decreased in rats with diet-induced obesity. In addition, changes in bacterial diversity were found in male Wistar rats fed a cafeteria diet for 15 weeks, which in turn were positively associated with total circulating levels of *n*-3 and *n*-7 and negatively with *n*-6/*n*-3 ratio in the two PL and CE fractions.

## 5. Conclusions

In the present study, we demonstrated that CAF diet induced obesity in male Wistar rats. This obesity phenotype was accompanied by increased energy intake and food efficiency. Characterization of the cafeteria diet indicated that it is rich in palmitic acid C16:0, stearic acid C18:0, oleic acid C18:1 *n*-9 and linoleic acid C18:2 *n*-6. The *n*-6/*n*-3 ratio of the cafeteria diet was 34:1. This indicates that a diet rich in omega-6 caused gut dysbiosis by decreasing bacterial diversity and relative abundance of *Bacteroidetes*, *Bacteroidia*, *Bacteroidales*, and *Prevotella*, as well as *TM7*, *TM7_3*, *CW040*, and *F16*. In contrast, an increase in the relative abundance of *Firmicutes*, *Bacilli*, *Lactobacillales*, *Lactobacillaceae* and *Lactobacillus* was found. These changes were associated with the obesity metabotype, finding an increase in the circulating levels of PL- SFAs, PL- C20:3 *n*-6, and CE C18:1 *n*-9 and a decrease in PL- C18:1 *n*-7 and PL- C18:3 *n*-3.

## Figures and Tables

**Table 1:** Fatty acid content (g/100 g dry matter) in the artisanal cafeteria diet.

Fatty Acids	Cafeteria Diet (g/100 g Dry Matter) *
Total saturated fatty acids (SFA)	7.94 ± 0.05
Caprylic acid C8:0	0.04 ± 0.00
Capric acid C10:0	0.03 ± 0.00
Lauric acid C12:0	0.03 ± 0.00
Myristic acid C14:0	0.18 ± 0.00
Pentadecanoic acid C15:0	0.02 ± 0.00
Palmitic acid C16:0	5.19 ± 0.04
Margaric acid C17:0	0.06 ± 0.00
Stearic acid C18:0	2.30 ± 0.02
Arachidic acid C20:0	0.06 ± 0.00
Behenic acid C22:0	0.02 ± 0.00
Lignoceric acid C24:0	0.01 ± 0.00
Total monounsaturated fatty acids (MUFA)	8.13 ± 0.082
Palmitoleic acid C16:1 *n*-7	0.31 ± 0.00
Heptadecenoic acid C17:1 *n*-7	0.03 ± 0.00
Oleic acid C18:1 *n*-9	7.21 ± 0.07
Vaccenic acid C18:1 *n*-7	0.46 ± 0.01
Eicosenoic acid C20:1 *n*-9	0.11 ± 0.00
Erucic acid C22:1 *n*-9	0.01 ± 0.00
Total polyunsaturated fatty acids (PUFA)	6.62 ± 0.14
Linoleic acid C18:2 *n*-6	6.29 ± 0.13
α-linolenic acid (ALA) C18:3 *n*-3	0.17 ± 0.00
Eicosadienoic acid C20:2 *n*-6	0.07 ± 0.00
Homo-γ-linoleic acid C20:3 *n*-6	0.01 ± 0.00
Dihomo-γ-linolenic acid C20:3 *n*-3	0.01 ± 0.00
Arachidonic acid C20:4 *n*-6	0.10 ± 0.00
Docosatetraenoic acid C22:4 *n*-6	0.02 ± 0.00
Docosapentaenoic acid (DPA) C22:5 *n*-3	0.01 ± 0.00
Total *n*-3	0.19 ± 0.00
Total *n*-6	6.49 ± 0.14
Total *n*-7	0.80 ± 0.01
Total *n*-9	7.33 ± 0.07
*n*-6/*n*-3 ratio	34.04 ± 0.29

Results are expressed as mean ± standard error (*n* = 4). *: grams/100 g of food dry weight.

**Table 2:** Food intake (g and kcal), food efficiency and biometric parameters in male Wistar rats fed a cafeteria diet.

Parameter	CTL	CAF
Daily food intake (g)	27.20 ± 0.42 ^a^	32.36 ± 1.23 ^b^
Daily caloric intake (kcal)	91.13 ± 1.42 ^a^	120.38 ± 4.59 ^b^
Food efficiency (g/100 kcal)	2.82 ± 0.03 ^a^	3.22 ± 0.12 ^b^
Liver weight (%)	2.83 ± 0.05 ^a^	2.50 ± 0.15 ^a^
Spleen weight (%)	0.17 ± 0.01 ^a^	0.12 ± 0.01 ^b^
sWAT (%)	1.23 ± 0.43 ^a^	1.28 ± 0.20 ^a^
eWAT (%)	2.75 ± 0.66 ^a^	2.63 ± 0.32 ^a^
rWAT (%)	2.87 ± 0.75 ^a^	3.29 ± 0.21 ^a^
mWAT (%)	2.11 ± 0.60 ^a^	1.63 ± 0.21 ^a^
BAT (%)	0.14 ± 0.03 ^a^	0.17 ± 0.02 ^a^
Gastrocnemius (%)	0.45 ± 0.03 ^a^	0.37 ± 0.02 ^a^
Soleus (%)	0.10 ± 0.01 ^a^	0.08 ± 0.01 ^a^

Results are expressed as mean ± standard error (*n* = 5 per group). Statistical analysis was performed using Student’s *t* test for two independent samples. Mean values with different letters (a,b) show significant differences (*p* < 0.05). CTL: control group; CAF: cafeteria group; sWAT: subcutaneous white adipose tissue; eWAT: epididymal white adipose tissue; rWAT: retroperitoneal white adipose tissue; mWAT: mesenteric white adipose tissue; BAT: brown adipose tissue.

**Table 3:** Relative abundance of gut bacterial taxa at order, family and genus levels of male Wistar rats.

Taxonomy	CTL	CAF
Phylum/Order		
*Actinobacteria*		
*Bifidobacteriales*	0.00 ± 0.00 ^a^	0.01 ± 0.01 ^a^
*Coriobacteriales*	0.02 ± 0.00 ^a^	0.03 ± 0.02 ^a^
*Bacteroidetes*		
*Bacteroidales*	53.82 ± 3.68 ^a^	39.89 ± 3.54 ^b^
*Cyanobacteria*		
*YS2*	0.80 ± 0.18 ^a^	0.92 ± 0.40 ^a^
*Elusimicrobia*		
*Elusimicrobiales*	0.21 ± 0.07 ^a^	0.34 ± 0.15 ^a^
*Firmicutes*		
*Clostridiale* s *	45.65 (23.47–45.87) ^a^	48.09 (28.72–53.56) ^a^
Erysipelotrichales *	0.04 (0.02–0.09) ^a^	0.25 (0.18–0.61) ^b^
*Lactobacillales*	1.48 ± 0.43 ^a^	8.82 ± 1.58 ^b^
*Turicibacterales*	0.27 ± 0.11 ^a^	0.24 ± 0.07 ^a^
*Proteobacteria*		
Burkholderiales *	0.22 (0.20–0.27) ^a^	0.60 (0.50–1.09) ^b^
Campylobacterales *	1.18 (0.89–3.47) ^a^	1.90 (0.21–9.24) ^a^
Desulfovibrionales *	0.43 (0.11–1.02) ^a^	0.29 (0.08–1.99) ^a^
Enterobacterales *	0.03 (0.01–0.07) ^a^	0.27 (0.00–0.35) ^a^
*Pasteurellales*	0.05 ± 0.01 ^a^	0.02 ± 0.01 ^b^
*RF32*	0.61 ± 0.33 ^a^	0.54 ± 0.20 ^a^
*Saccharibacteria (TM7)*		
*CW040*	0.64 ± 0.05 ^a^	0.26 ± 0.03 ^b^
*Tenericutes*		
*ML615J*-*28*	0.01 ± 0.01 ^a^	0.00 ± 0.00 ^a^
RF39 *	0.00 (0.00–0.01) ^a^	0.00 (0.00–0.24) ^a^
Phylum/Family		
*Actinobacteria*		
*Bifidobacteriaceae*	0.00 ± 0.00 ^a^	0.01 ± 0.01 ^a^
*Coriobacteriaceae*	0.02 ± 0.01 ^a^	0.03 ± 0.02 ^a^
*Micrococcaceae*	0.01 ± 0.00 ^a^	0.00 ± 0.00 ^a^
*Bacteroidetes*		
*Bacteroidaceae*	6.43 ± 0.70 ^a^	8.43 ± 0.49 ^b^
*Barnesiellaceae*	0.00 ± 0.00 ^a^	0.01 ± 0.01 ^b^
*Dehalobacteriaceae*	0.22 ± 0.04 ^a^	0.19 ± 0.07 ^a^
*Odoribacteraceae*	0.17 ± 0.03 ^a^	0.25 ± 0.05 ^a^
*Paraprevotellaceae*	9.41 ± 1.79 ^a^	8.54 ± 1.92 ^a^
*Porphyromonadaceae*	1.60 ± 0.29 ^a^	2.28 ± 0.18 ^a^
Prevotellaceae *	18.86 (7.37–19.91) ^a^	6.80 (2.49–12.39) ^b^
*Rikenellaceae*	1.07 ± 0.21 ^a^	0.46 ± 0.08 ^b^
*S24*-*7* (*Muribaculaceae*) *	21.64 (20.48–36.65) ^a^	20.14 (14.95–20.92) ^b^
*Elusimicrobia*		
*Elusimicrobiaceae*	0.24 ± 0.08 ^a^	0.39 ± 0.17 ^a^
*Euryarchaeota*		
*Methanobacteriaceae*	0.03 ± 0.03 ^a^	0.02 ± 0.01 ^a^
*Firmicutes*		
*Christensenellaceae*	0.07 ± 0.01 ^a^	0.16 ± 0.05 ^a^
*Clostridiaceae*	1.75 ± 0.74 ^a^	1.29 ± 0.24 ^a^
Erysipelotrichaceae *	0.04 (0.02–0.10) ^a^	0.28 (0.19–0.71) ^b^
*Lachnospiraceae*	7.97 ± 1.63 ^a^	6.22 ± 0.60 ^a^
*Lactobacillaceae*	1.65 ± 0.47 ^a^	9.91 ± 1.70 ^b^
*Mogibacteriaceae*	0.26 ± 0.03 ^a^	0.27 ± 0.05 ^a^
*Peptococcaceae*	0.08 ± 0.03 ^a^	0.16 ± 0.03 ^a^
*Ruminococcaceae*	23.45 ± 2.11 ^a^	30.17 ± 4.81 ^a^
*Streptococcaceae*	0.00 ± 0.00 ^a^	0.01 ± 0.01 ^a^
*Turicibacteraceae*	0.30 ± 0.13 ^a^	0.27 ± 0.08 ^a^
*Veillonellaceae*	0.03 ± 0.01 ^a^	0.01 ± 0.00 ^a^
*Proteobacteria*		
*Alcaligenaceae*	0.26 ± 0.01 ^a^	0.87 ± 0.16 ^b^
Desulfovibrionaceae *	0.50 (0.12–1.18) ^a^	0.31 (0.09–2.27) ^a^
Enterobacteriaceae *	0.03 (0.01–0.07) ^a^	0.03 (0.00–0.38) ^a^
*Helicobacteraceae*	1.76 ± 0.57 ^a^	3.50 ± 1.82 ^a^
*Pasteurellaceae*	0.06 ± 0.01 ^a^	0.02 ± 0.01 ^b^
*Saccharibacteria (TM7)*		
*F16*	0.72 ± 0.05 ^a^	0.30 ± 0.03 ^b^
Phylum/Genus		
*Actinobacteria*		
*Adlercreutzia*	0.01 ± 0.00 ^a^	0.01 ± 0.00 ^a^
*Bifidobacterium*	0.00 ± 0.00 ^a^	0.02 ± 0.01 ^a^
*Rothia*	0.01 ± 0.00 ^a^	0.01 ± 0.00 ^a^
*Bacteroidetes*		
*Bacteroides*	11.75 ± 1.70 ^a^	14.05 ± 0.65 ^a^
*Butyricimonas*	0.29 ± 0.05 ^a^	0.42 ± 0.07 ^a^
*CF231*	16.91 ± 3.21 ^a^	14.26 ± 3.44 ^a^
*Parabacteroides*	2.88 ± 0.56 ^a^	3.83 ± 0.35 ^a^
Prevotella *	32.99 (14.91–35.32) ^a^	12.44 (4.13–22.16) ^b^
*Prevotella_2*	0.02 ± 0.01 ^a^	0.01 ± 0.01 ^a^
*Firmicutes*		
Allobaculum *	0.02 (0.00–0.14) ^a^	0.35 (0.23–1.24) ^b^
Anaerostipes *	0.02 (0.01–0.13) ^a^	0.00 (0.00–0.03) ^b^
Anaerotruncus *	0.00 (0.00–0.01) ^a^	0.04 (0.03–0.06) ^b^
*Blautia*	0.75 ± 0.35 ^a^	1.67 ± 0.39 ^a^
Butyricicoccus *	0.04 (0.01–1.26) ^a^	0.32 (0.11–0.48) ^a^
*Clostridium*	2.39 ± 1.16 ^a^	1.18 ± 0.26 ^a^
Clostridium_2 *	0.10 (0.00–0.53) ^a^	0.00 (0.00–0.01) ^b^
*Coprococcus*	2.71 ± 0.58 ^a^	1.34 ± 0.46 ^a^
*Dehalobacterium*	0.39 ± 0.08 ^a^	0.33 ± 0.12 ^a^
*Dorea*	0.82 ± 0.65 ^a^	0.72 ± 0.15 ^a^
*Eubacterium*	0.00 ± 0.00 ^a^	0.02 ± 0.01 ^b^
*Faecalibacterium*	0.17 ± 0.06 ^a^	1.87 ± 0.91 ^b^
*Lactobacillus*	2.97 ± 0.86 ^a^	16.21 ± 2.29 ^b^
*Oscillospira*	16.46 ± 2.15 ^a^	7.98 ± 1.09 ^b^
*Roseburia*	0.21 ± 0.10 ^a^	0.37 ± 0.17 ^a^
Ruminococcus *	0.24 (0.16–0.43) ^a^	0.05 (0.01–1.26) ^a^
*Ruminococcus_2*	8.97 ± 1.22 ^a^	19.53 ± 4.72 ^a^
*SMB53*	0.66 ± 0.40 ^a^	0.83 ± 0.27 ^a^
*Streptococcus*	0.01 ± 0.00 ^a^	0.02 ± 0.01 ^a^
*Turicibacter*	0.57 ± 0.26 ^a^	0.47 ± 0.15 ^a^
*Veillonella*	0.05 ± 0.01 ^a^	0.02 ± 0.01 ^a^
*Proteobacteria*		
*Aggregatibacter*	0.11 ± 0.02 ^a^	0.03 ± 0.01 ^b^
*Desulfovibrio*	0.10 ± 0.04 ^a^	0.11 ± 0.03 ^a^
Escherichia *	0.06 (0.03–0.14) ^a^	0.03 (0.00–0.06) ^a^
*Helicobacter*	0.07 ± 0.07 ^a^	0.00 ± 0.00 ^a^
*Sutterella*	0.46 ± 0.02 ^a^	1.45 ± 0.28 ^b^

*: These gut bacterial taxa are presented as median (interquartile range). All other bacteria are presented as mean ± standard error (*n* = 5). Statistical analysis was performed using Student’s *t* test for two independent samples or its non-parametric equivalent, Mann-Whitney U test. Mean values with different letters (a,b) show significant differences (*p* < 0.05). CTL: control group; CAF: cafeteria group.

**Table 4:** Serum fatty acid profiles of phospholipid fraction of male Wistar rats.

Fatty Acids	CTL (%) *	CAF (%) *
Total saturated fatty acids (SFA)	56.61 ± 0.89 ^a^	57.73 ± 1.52 ^a^
Caprylic acid C8:0	2.71 ± 0.39 ^a^	4.16 ± 0.75 ^a^
Capric acid C10:0	1.64 ± 0.54 ^a^	1.58 ± 0.80 ^a^
Lauric acid C12:0	1.83 ± 0.53 ^a^	2.46 ± 0.43 ^a^
Myristic acid C14:0	0.85 ± 0.13 ^a^	1.26 ± 0.24 ^a^
Pentadecanoic acid C15:0	0.42 ± 0.12 ^a^	0.36 ± 0.15 ^a^
Palmitic acid C16:0	25.32 ± 1.54 ^a^	23.72 ± 1.17 ^a^
Stearic acid C18:0	22.53 ± 0.83 ^a^	22.39 ± 1.15 ^a^
Araquidic acid C20:0	0.18 ± 0.02 ^a^	0.25 ± 0.07 ^a^
Behenic acid C22:0	0.28 ± 0.01 ^a^	0.35 ± 0.04 ^a^
Lignoceric acid C24:0	0.84 ± 0.12 ^a^	1.20 ± 0.20 ^a^
Total monounsaturated fatty acids (MUFA)	6.08 ± 0.77 ^a^	5.74 ± 0.72 ^a^
Palmitoleic acid C16:1 *n*-7	0.43 ± 0.11 ^a^	0.31 ± 0.06 ^a^
Oleic acid C18:1 *n*-9	3.41 ± 0.66 ^a^	4.07 ± 0.65 ^a^
Vaccenic acid C18:1 *n*-7	1.85 ± 0.09 ^a^	0.79 ± 0.03 ^b^
Nervonic acid C24:1 *n*-9	0.39 ± 0.03 ^a^	0.50 ± 0.08 ^b^
Total polyunsaturated fatty acids (PUFA)	37.31 ± 1.58 ^a^	36.53 ± 0.99 ^a^
Linoleic acid C18:2 *n*-6	8.92 ± 0.46 ^a^	9.11 ± 0.80 ^a^
γ-linolenic acid (GLA) C18:3 *n*-6	1.71 ± 0.37 ^a^	2.39 ± 0.43 ^a^
α-linolenic acid (ALA) C18:3 *n*-3	0.23 ± 0.03 ^a^	0.15 ± 0.03 ^b^
Eicosadienoic acid C20:2 *n*-6	0.35 ± 0.07 ^a^	0.20 ± 0.02 ^b^
Dihomo-γ-linoleic acid C20:3 *n*-6	0.36 ± 0.06 ^a^	1.91 ± 0.38 ^b^
Arachidonic acid C20:4 *n*-6	18.68 ± 0.98 ^a^	17.11 ± 1.00 ^a^
Eicosapentaenoic acid C20:5 *n*-3	0.69 ± 0.18 ^a^	1.30 ± 0.12 ^b^
Docosapentaenoic acid C22:5 *n*-3	0.78 ± 0.10 ^a^	1.47 ± 0.07 ^b^
Docosahexanoic acid C22:6 *n*-3	5.58 ± 1.19 ^a^	2.87 ± 0.18 ^b^
Total *n*-3	7.28 ± 1.37 ^a^	5. 80 ± 0.27 ^a^
Total *n*-6	30.02 ± 1.63 ^a^	30.73 ± 0.81 ^a^
Total *n*-7	2.27 ± 0.14 ^a^	1.09 ± 0.08 ^b^
Total *n*-9	3.80 ± 0.66 ^a^	4.64 ± 0.70 ^a^
*n*-6/*n*-3 ratio	4.61 ± 0.69 ^a^	5.34 ± 0.22 ^a^

Results are expressed as mean ± standard error (*n* = 5 per group). Statistical analysis was performed using Student’s *t* test for two independent samples. Mean values with different letters (a,b) show significant differences (*p* < 0.05). CTL: control group; CAF: cafeteria group. *: Fatty acids are expressed as a percentage of total FA composition.

**Table 5:** Serum fatty acid profiles of cholesteryl ester fraction of male Wistar rats.

Fatty Acids	CTL (%) *	CAF (%) *
Total saturated fatty acids (SFA)	51.33 ± 2.07 ^a^	52.24 ± 4.08 ^a^
Caprylic acid C8:0	6.66 ± 1.24 ^a^	7. 30 ± 1.09 ^a^
Capric acid C10:0	9.08 ± 0.85 ^a^	4.34 ± 0.45 ^b^
Lauric acid C12:0	8.67 ± 0.83 ^a^	5.55 ± 0.87 ^b^
Myristic acid C14:0	0.81 ± 0.06 ^a^	0.63 ± 0.06 ^a^
Palmitic acid C16:0	14.70 ± 1.86 ^a^	18.57 ± 2.69 ^a^
Stearic acid C18:0	11.38 ± 1.60 ^a^	15.85 ± 3.35 ^a^
Total monounsaturated fatty acids (MUFA)	11.10 ± 0.20 ^a^	13.28 ± 2.26 ^a^
Myristotelic acid C14:1 *n*-5	1.68 ± 0.45 ^a^	1.13 ± 0.31 ^a^
Palmitoleic acid C16:1 *n*-7	1.25 ± 0.08 ^a^	0.76 ± 0.11 ^b^
Oleic acid C18:1 *n*-9	6.59 ± 0.67 ^a^	10.52 ± 1.83 ^b^
Vaccenic acid C18:1 *n*-7	1.58 ± 0.20 ^a^	0.89 ± 0.05 ^b^
Total polyunsaturated fatty acids (PUFA)	37.57 ± 2.02 ^a^	34.48 ± 1.96 ^a^
Linoleic acid C18:2 *n*-6	9.66 ± 2.08 ^a^	16.96 ± 2.16 ^b^
α-linolenic acid (ALA) C18:3 *n*-3	1.00 ± 0.14 ^a^	0.37 ± 0.08 ^b^
Dihomo-γ-linoleic acid C20:3 *n*-6	3.86 ± 1.46 ^a^	0.42 ± 0.05 ^b^
Arachidonic acid C20:4 *n*-6	22.48 ± 1.57 ^a^	16.32 ± 0.96 ^b^
Eicosapentaenoic acid C20:5 *n*-3	0.56 ± 0.07 ^a^	0.40 ± 0.10 ^a^
Total *n*-3	1.56 ± 0.19 ^a^	0.77 ± 0.17 ^b^
Total *n*-6	36.00 ± 1.97 ^a^	33.70 ± 1.82 ^a^
Total *n*-7	2.82 ± 0.25 ^a^	1.63 ± 0.15 ^b^
Total *n*-9	6.59 ± 0.67 ^a^	10.52 ± 1.83 ^a^
*n*-6/*n*-3 ratio	24.93 ± 3.88 ^a^	56.37 ± 15.80 ^a^

Results are expressed as mean ± standard error (*n* = 5 per group). Statistical analysis was performed using Student’s *t* test for two independent samples. Mean values with different letters (a,b) show significant differences (*p* < 0.05). CTL: control group; CAF: cafeteria group. *: Fatty acids are expressed as a percentage of total FA composition.

## Data Availability

Data are contained within this article.

## References

[1] Kearney J. (2010). Food Consumption Trends and Drivers. Philos. Trans. R. Soc. Lond. B. Biol. Sci..

[2] Zinöcker M.K., Lindseth I.A. (2018). The Western Diet-Microbiome-Host Interaction and Its Role in Metabolic Disease. Nutrients.

[3] Crovesy L., Rosado E.L. (2019). Interaction between Genes Involved in Energy Intake Regulation and Diet in Obesity. Nutrition.

[4] Pruszyńska-Oszmałek E., Wojciechowska M., Sassek M., Krauss H., Leciejewska N., Szczepankiewicz D., Ślósarz P., Nogowski L., Kołodziejski P.A. (2021). The Long-Term Effects of High-Fat and High-Protein Diets on the Metabolic and Endocrine Activity of Adipocytes in Rats. Biology.

[5] Lalanza J.F., Snoeren E.M.S. (2021). The Cafeteria Diet: A Standardized Protocol and Its Effects on Behavior. Neurosci. Biobehav. Rev..

[6] Ribaroff G.A., Wastnedge E., Drake A.J., Sharpe R.M., Chambers T.J.G. (2017). Animal Models of Maternal High Fat Diet Exposure and Effects on Metabolism in Offspring: A Meta-Regression Analysis. Obes. Rev..

[7] Martínez-Micaelo N., González-Abuín N., Terra X., Ardévol A., Pinent M., Petretto E., Behmoaras J., Blay M. (2016). Identification of a Nutrient-Sensing Transcriptional Network in Monocytes by Using Inbred Rat Models on a Cafeteria Diet. Dis. Model. Mech..

[8] de la Garza A.L., Treviño-de Alba C., Cárdenas-Pérez R.E., Camacho A., Gutierrez-Lopez M., Castro H., Vinciguerra M., Cordero Sanchez P. (2021). Chapter 6—Fatty Acid Intake during Perinatal Periods.

[9] Simopoulos A.P. (2016). An Increase in the Omega-6/Omega-3 Fatty Acid Ratio Increases the Risk for Obesity. Nutrients.

[10] DiNicolantonio J.J., O’Keefe J. (2021). The Importance of Maintaining a Low Omega-6/Omega-3 Ratio for Reducing the Risk of Autoimmune Diseases, Asthma, and Allergies. Mo. Med..

[11] Ghosh S., Molcan E., DeCoffe D., Dai C., Gibson D.L. (2013). Diets Rich in N-6 PUFA Induce Intestinal Microbial Dysbiosis in Aged Mice. Br. J. Nutr..

[12] Machate D.J., Figueiredo P.S., Marcelino G., Guimarães R.D.C.A., Hiane P.A., Bogo D., Pinheiro V.A.Z., Oliveira L.C.S.d., Pott A. (2020). Fatty Acid Diets: Regulation of Gut Microbiota Composition and Obesity and Its Related Metabolic Dysbiosis. Int. J. Mol. Sci..

[13] Magne F., Gotteland M., Gauthier L., Zazueta A., Pesoa S., Navarrete P., Balamurugan R. (2020). The Firmicutes/Bacteroidetes Ratio: A Relevant Marker of Gut Dysbiosis in Obese Patients?. Nutrients.

[14] Alemao C.A., Budden K.F., Gomez H.M., Rehman S.F., Marshall J.E., Shukla S.D., Donovan C., Forster S.C., Yang I.A., Keely S. (2021). Impact of Diet and the Bacterial Microbiome on the Mucous Barrier and Immune Disorders. Allergy.

[15] Zhang X., Coker O.O., Chu E.S., Fu K., Lau H.C.H., Wang Y.-X., Chan A.W.H., Wei H., Yang X., Sung J.J.Y. (2021). Dietary Cholesterol Drives Fatty Liver-Associated Liver Cancer by Modulating Gut Microbiota and Metabolites. Gut.

[16] Pedreschi R., Hollak S., Harkema H., Otma E., Robledo P., Westra E., Somhorst D., Ferreyra R., Defilippi B.G. (2016). Impact of Postharvest Ripening Strategies on ‘Hass’ Avocado Fatty Acid Profiles. S. Afr. J. Bot..

[17] Castillo E.C., Elizondo-Montemayor L., Hernández-Brenes C., Rodríguez-Sánchez D.G., Silva-Platas C., Marín-Obispo L.M., Rodríguez-Gutierrez N.A., Treviño V., García-Rivas G. (2020). Integrative Analysis of Lipid Profiles in Plasma Allows Cardiometabolic Risk Factor Clustering in Children with Metabolically Unhealthy Obesity. Oxid. Med. Cell. Longev..

[18] Etxeberria U., De La Garza A.L., Alfredo Martínez J., Milagro F.I. (2015). Biocompounds Attenuating the Development of Obesity and Insulin Resistance Produced by a High-Fat Sucrose Diet. Nat. Prod. Commun..

[19] de la Garza A.L., Romero-Delgado B., Martínez-Tamez A.M., Cárdenas-Tueme M., Camacho-Zamora B.D., Matta-Yee-Chig D., Sánchez-Tapia M., Torres N., Camacho-Morales A. (2022). Maternal Sweeteners Intake Modulates Gut Microbiota and Exacerbates Learning and Memory Processes in Adult Male Offspring. Front. Pediatr..

[20] Rakhra V., Galappaththy S.L., Bulchandani S., Cabandugama P.K. (2020). Obesity and the Western Diet: How We Got Here. Mo. Med..

[21] Gual-Grau A., Guirro M., Mayneris-Perxachs J., Arola L., Boqué N. (2019). Impact of Different Hypercaloric Diets on Obesity Features in Rats: A Metagenomics and Metabolomics Integrative Approach. J. Nutr. Biochem..

[22] Boqué N., Campión J., de la Iglesia R., de la Garza A.L., Milagro F.I., San Román B., Bañuelos O., Martínez J.A. (2013). Screening of Polyphenolic Plant Extracts for Anti-Obesity Properties in Wistar Rats. J. Sci. Food Agric..

[23] Cardenas-Perez R.E., Fuentes-Mera L., De La Garza A.L., Torre-Villalvazo I., Reyes-Castro L.A., Rodriguez-Rocha H., Garcia-Garcia A., Corona-Castillo J.C., Tovar A.R., Zambrano E. (2018). Maternal Overnutrition by Hypercaloric Diets Programs Hypothalamic Mitochondrial Fusion and Metabolic Dysfunction in Rat Male Offspring. Nutr. Metab..

[24] Monteiro C.A., Cannon G., Levy R.B., Moubarac J.-C., Louzada M.L.C., Rauber F., Khandpur N., Cediel G., Neri D., Martinez-Steele E. (2019). Ultra-Processed Foods: What They Are and How to Identify Them. Public Health Nutr..

[25] Mayerhofer A., Dietrich K.-G., Urbanski H.F., Köhn F.-M., Pickl U., Trottmann M., Kievit P., Welter H. (2020). Palmitic Acid Targets Human Testicular Peritubular Cells and Causes a Pro-Inflammatory Response. J. Clin. Med..

[26] Saraswathi V., Kumar N., Gopal T., Bhatt S., Ai W., Ma C., Talmon G.A., Desouza C. (2020). Lauric Acid versus Palmitic Acid: Effects on Adipose Tissue Inflammation, Insulin Resistance, and Non-Alcoholic Fatty Liver Disease in Obesity. Biology.

[27] Hua M.-C., Su H.-M., Lai M.-W., Yao T.-C., Tsai M.-H., Liao S.-L., Lai S.-H., Huang J.-L. (2021). Palmitoleic and Dihomo-γ-Linolenic Acids Are Positively Associated with Abdominal Obesity and Increased Metabolic Risk in Children. Front. Pediatr..

[28] Bermúdez-Cardona J., Velásquez-Rodríguez C. (2016). Profile of Free Fatty Acids and Fractions of Phospholipids, Cholesterol Esters, and Triglycerides in Serum of Obese Youth with and without Metabolic Syndrome. Nutrients.

[29] Sampath H., Ntambi J.M. (2005). The Fate and Intermediary Metabolism of Stearic Acid. Lipids.

[30] Johnston L.W., Harris S.B., Retnakaran R., Zinman B., Giacca A., Liu Z., Bazinet R.P., Hanley A.J. (2016). Longitudinal Associations of Phospholipid and Cholesteryl Ester Fatty Acids with Disorders Underlying Diabetes. J. Clin. Endocrinol. Metab..

[31] Noble E.E., Hsu T.M., Kanoski S.E. (2017). Gut to brain dysbiosis: Mechanisms linking western diet consumption, the microbiome, and cognitive impairment. Front. Behav. Neurosci..

[32] Li H., Liu Y., Zhang X., Xu Q., Zhang Y., Xue C., Guo C. (2018). Medium-Chain Fatty Acids Decrease Serum Cholesterol via Reduction of Intestinal Bile Acid Reabsorption in C57BL/6J Mice. Nutr. Metab..

[33] Dekkers K.F., Sayols-Baixeras S., Baldanzi G., Nowak C., Hammar U., Nguyen D., Varotsis G., Brunkwall L., Nielsen N., Eklund A.C. (2022). An Online Atlas of Human Plasma Metabolite Signatures of Gut Microbiome Composition. Nat. Commun..

[34] de Wit N.J.W., Derrien M., Bosch-Vermeulen H., Oosterink E., Keshtkar S., Duval C., de Vogel-van den Bosch J., Kleerebezem M., Müller M., van der Meer R. (2012). Saturated Fat Stimulates Obesity and Hepatic Steatosis and Affects Gut Microbiota Composition by an Enhanced Overflow of Dietary Fat to the Distal Intestine. Am. J. Physiol. Gastrointest. Liver Physiol..

[35] Etxeberria U., Milagro F., González- Navarro C.J., Alfredo Martínez J. (2016). Role of Gut Microbiota in Obesity. An. Real Acad. Farm..

[36] Gomes A.C., Hoffmann C., Mota J.F. (2020). Gut Microbiota Is Associated with Adiposity Markers and Probiotics May Impact Specific Genera. Eur. J. Nutr..

[37] Hu Y., Xu J., Sheng Y., Liu J., Li H., Guo M., Xu W., Luo Y., Huang K., He X. (2022). Pleurotus Ostreatus Ameliorates Obesity by Modulating the Gut Microbiota in Obese Mice Induced by High-Fat Diet. Nutrients.

[38] Duckett S.K., Volpi-Lagreca G., Alende M., Long N.M. (2014). Palmitoleic Acid Reduces Intramuscular Lipid and Restores Insulin Sensitivity in Obese Sheep. Diabetes Metab. Syndr. Obes..

[39] Bailén M., Bressa C., Martínez-López S., González-Soltero R., Montalvo Lominchar M.G., San Juan C., Larrosa M. (2020). Microbiota Features Associated with a High-Fat/Low-Fiber Diet in Healthy Adults. Front. Nutr..

[40] Myles I.A., Fontecilla N.M., Janelsins B.M., Vithayathil P.J., Segre J.A., Datta S.K. (2013). Parental Dietary Fat Intake Alters Offspring Microbiome and Immunity. J. Immunol..

[41] Tseng C.-H., Wu C.-Y. (2019). The Gut Microbiome in Obesity. J. Formos. Med. Assoc..

[42] Requena T., Martínez-Cuesta M.C., Peláez C. (2018). Diet and Microbiota Linked in Health and Disease. Food Funct..

[43] Pu S., Khazanehei H., Jones P.J., Khafipour E. (2016). Interactions between Obesity Status and Dietary Intake of Monounsaturated and Polyunsaturated Oils on Human Gut Microbiome Profiles in the Canola Oil Multicenter Intervention Trial (COMIT). Front. Microbiol..

